# Probing the non-equilibrium transient state in magnetite by a jitter-free two-color X-ray pump and X-ray probe experiment

**DOI:** 10.1063/1.5042847

**Published:** 2018-09-26

**Authors:** N. Pontius, M. Beye, C. Trabant, R. Mitzner, F. Sorgenfrei, T. Kachel, M. Wöstmann, S. Roling, H. Zacharias, R. Ivanov, R. Treusch, M. Buchholz, P. Metcalf, C. Schüßler-Langeheine, A. Föhlisch

**Affiliations:** 1 Institut für Methoden und Instrumentierung der Forschung mit Synchrotronstrahlung, Helmholtz-Zentrum Berlin für Materialien und Energie, Albert-Einstein-Str. 15, 12489 Berlin, Germany; 2 WWU Münster, Physikalisches Institut, Wilhelm-Klemm-Str. 10, 48149 Münster, Germany; 3 Deutsches Elektronen-Synchrotron, Notkestr. 85, 22607 Hamburg, Germany; 4 II. Physikalisches Institut, Universität zu Köln, Zülpicher Str. 77, 50937 Köln, Germany; 5 School of Materials Engineering, Purdue University, West Lafayette, Indiana 47907, USA

## Abstract

We present a general experimental concept for jitter-free pump and probe experiments at free electron lasers. By generating pump and probe pulse from one and the same X-ray pulse using an optical split-and-delay unit, we obtain a temporal resolution that is limited only by the X-ray pulse lengths. In a two-color X-ray pump and X-ray probe experiment with sub 70 fs temporal resolution, we selectively probe the response of orbital and charge degree of freedom in the prototypical functional oxide magnetite after photoexcitation. We find electronic order to be quenched on a time scale of (30 ± 30) fs and hence most likely faster than what is to be expected for any lattice dynamics. Our experimental result hints to the formation of a short lived transient state with decoupled electronic and lattice degree of freedom in magnetite. The excitation and relaxation mechanism for X-ray pumping is discussed within a simple model leading to the conclusion that within the first 10 fs the original photoexcitation decays into low-energy electronic excitations comparable to what is achieved by optical pump pulse excitation. Our findings show on which time scales dynamical decoupling of degrees of freedom in functional oxides can be expected and how to probe this selectively with soft X-ray pulses. Results can be expected to provide crucial information for theories for ultrafast behavior of materials and help to develop concepts for novel switching devices.

## I. INTRODUCTION

The energy scales of the coupling between spin, orbital, and structural degrees of freedom (DOF) in functional solids imply that correlated dynamics in such materials occurs on femtosecond time scales. Only with suitable temporal resolution in a pump and probe experiment, one can thus hope to observe a transient decoupling of different DOFs and to disentangle their respective role in, e.g., phase transitions, potentially enabling the discovery of new switching functionalities.

Creating novel material properties through the generation of non-equilibrium states in functional solids is an approach that has been pursued intensively in the recent decades.^1–5^ In thermal equilibrium, the electronic, magnetic, orbital, and structural degrees of freedom (DOF) are intimately linked. Given the individual lifetimes of particular subsystem excitations and couplings, this link should transiently break under non-equilibrium conditions. Such transient states are interesting as they may be associated with macroscopic properties linked to functionality. Furthermore, novel active coordinates may form that can overcome energy barriers to a different thermodynamic phase. Both possibilities promise insights to make switching processes for information technology and storage applications faster and more energy efficient. However, such approaches require the development of experimental techniques to excite transient states with decoupled DOF and study their properties during their ultrashort lifetime with sufficient selectivity and sensitivity.

Femtosecond time-resolved methods, which are being developed to tackle this goal, in general, have to meet two requirements. On the one hand, the temporal resolution needs to be sufficiently high while, on the other hand, the method should also enable to probe the various DOF selectively. Resonant X-ray techniques are established as powerful tools to selectively probe electronic and spin ordering phenomena as well as structural symmetry. With the advent of free-electron lasers (FELs) and other short X-ray pulse sources with tunable photon energy,^6–10^ these methods can now be applied with femtosecond time-resolution. Typically, these studies require the synchronization of fs-optical laser systems for sample excitation with the probing X-ray source. Over the years, the temporal jitter of the synchronization has become smaller, but it remains challenging to reach temporal resolutions well below 100 fs in routine operation. Even when the jitter is corrected for by elaborate parallel single-shot cross-correlation measurements,^11,12^ the time resolution is often limited to around 100 fs due to the combination of pulse lengths limitations in the FEL and available optical laser system and residual uncorrected transport jitter between those light sources often placed hundreds of meters apart.

In this contribution, we report on an experimental scheme that we successfully used at the FEL FLASH (Free-Electron Laser in Hamburg) and that allows for an *a priori* jitter-free pump and probe X-ray experiment. In this experimental setup, we use one and the same FEL photon pulse for pumping as well as for probing. The pump and the probe part of the pulses—in our case the fundamental and third harmonic component of the same FEL pulse, respectively—are separated by a variable time delay with an opto-mechanical split-and-delay line.^13^ The delay between the two pulses is given by the geometrical path lengths of the pump and probe branches. The temporal resolution of the X-ray cross-correlation setup is then only limited by the FEL pulse lengths.

We apply this method to investigate the photoinduced transient state preceding the driven, non-equilibrium insulator-to-metal transition in the transition metal oxide magnetite (Fe_3_O_4_).^14^ In equilibrium, upon cooling below 123 K, magnetite's electrical conductivity drops by two orders of magnitude in a first order phase transition named after Verwey.^15^ The Verwey transition involves a structural transition from the cubic high-temperature to the monoclinic low-temperature symmetry^16^ as well as the formation of a complex spatial modulation of electronic states. For the latter, different models have been put forward, some of which include charge order (a spatial pattern of electron-poor and electron-rich ions) on the octahedrally coordinated Fe ions sites.^16^ Common to all models is the appearance of orbital order (OO), i.e., the occupation of different orbitals on different sites.^17–21^ The detailed character of this electronic-state modulation is so far unclear. The same applies to the mechanism of the Verwey transition, in particular, the role of the different degrees of freedom. Our results shed light on this latter aspect.

Electronic and lattice DOF can be studied by resonant and non-resonant X-ray diffraction.^16,20,22–25^ Their non-equilibrium dynamics can be addressed in pump and probe diffraction experiments. In a first attempt to disentangle electronic and structural dynamics in magnetite, we used infrared laser pulses to drive the electronic sector out of the low-temperature phase and X-ray pulses from the free-electron-lasers FLASH^24^ and LCLS (Linac Coherent Light Source)^23^ to probe the response. In both cases, the temporal resolution was limited by jitter between externally synchronized optical pump lasers and probe (FEL) sources. Within the resulting 300 fs resolution at LCLS, we found electronic order and lattice distortion to identically respond in speed and amplitude without any hint of decoupling. Generally, though, one expects lattice dynamics to be slower than electronic dynamics. The fact that we observed no difference indicates that lattice dynamics occurs on time scales well below the 300 fs temporal resolution. Indeed, sub-300 fs structural dynamics have also been found in other transition-metal oxides.^26,27^ For the structural part of the Verwey transition, the dominant Δ_5_ und *X*_3_ phonons^28,29^ allow us to estimate a structural relaxation time, which we assume to be at least of the order of one quarter of their phonon oscillation period, yielding ≥ 105 fs and ≥ 65 fs, respectively.^30^ These time scales also define the temporal resolution needed to observe dynamical decoupling.

## II. EXPERIMENTAL DETAILS

**FIG. 1.**
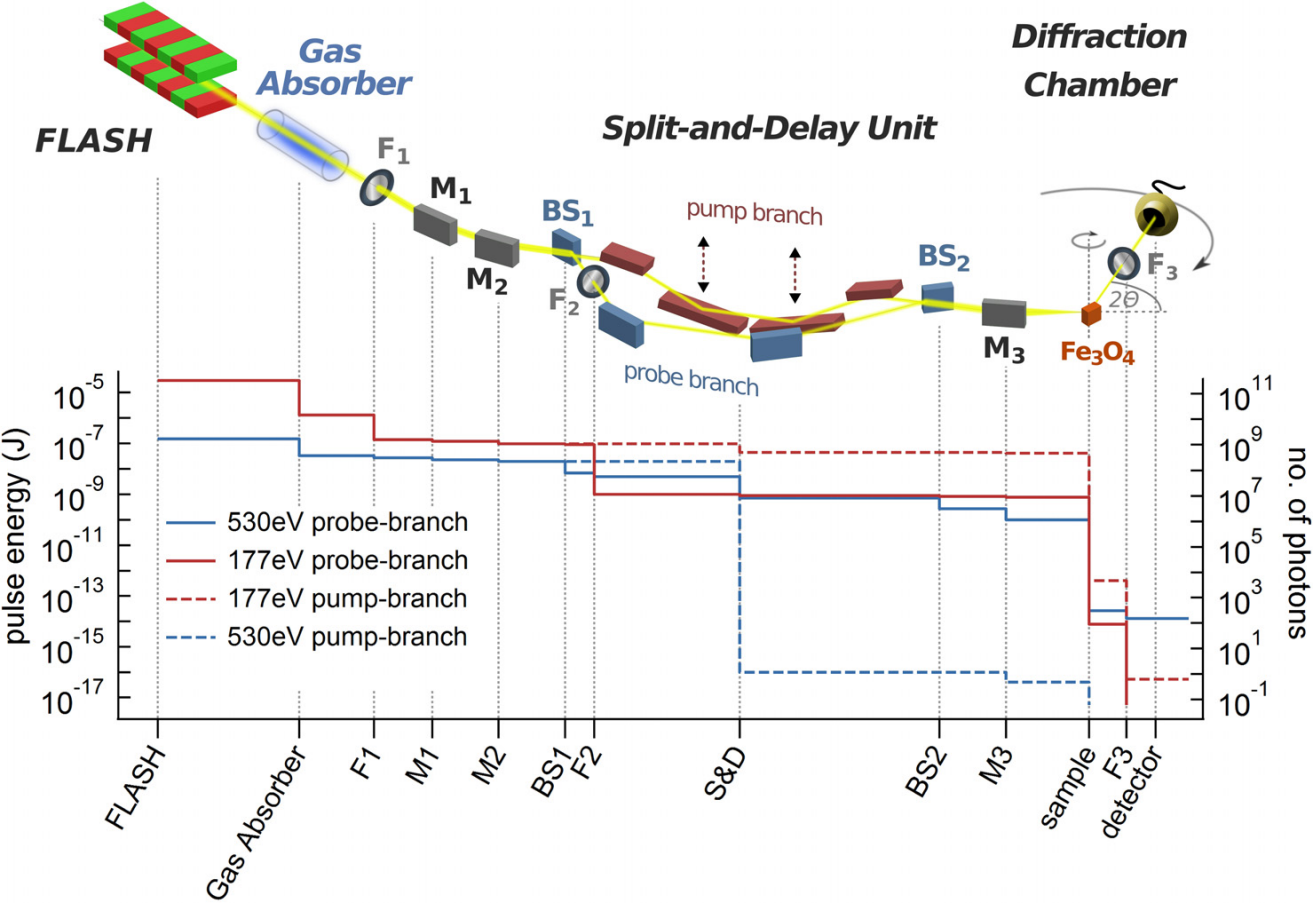
Two-color X-ray pump and probe scheme at the BL2-beamline at FLASH. The FEL undulator generates fs X-ray pulses at fundamental (177 eV) and third harmonics (530 eV) photon energies. The split-and-delay unit^13^ serves to separate pump (fundamental) and probe (third harmonics) pulses. The graph gives the X-ray pulse energies (number of photons on the right hand scale) of pump (red dashed line) and probe pulses (blue solid) at any position of the setup. The solid red and the dashed blue lines are the pulse energies of fundamental and third harmonics photons in the respective other branches which are negligible for excitation and detection, respectively. The beam path contains beamline mirrors M_1_–M_3_, Aluminum filters F_1_–F_3_, and beam splitting mirrors BS_1_ and BS_2_ of the split-and-delay unit.

For probing electronic order, we use photons of 530 eV, corresponding to the oxygen 1s→2p (K) transition in the third FEL harmonic. Since Iron and Oxygen states in magnetite hybridize, resonant diffraction at the oxygen-*K*-resonance has been shown to be a sensitive probe for electronic order on the Fe-sites.^22,31^ For pumping, we use the FEL fundamental at 177 eV. Photoexcitation occurs non-resonantly via fast non-radiative decay of the initial excitation^32–34^ as discussed in more detail below. To reach X-ray pulse-length limited jitter-free temporal resolution, we used the setup shown in Fig. 1. With the split-and-delay unit (SDU) at beamline BL2 of the FEL FLASH, we spatially split a single X-ray pulse into two equal parts. The two pulse components propagate through the SDU on two different paths. By varying the path-lengths, the time delay between both pulses can be tuned.^13^ Because of the different deflection angles on the mirrors of the two paths and an additional Al-filter (F_2_) in the probe branch, the pulse-component used for pumping is dominated by the fundamental X-ray photon energy of 177 eV, while that for probing mostly consists of the third FEL harmonic at 530 eV. The two beam components leave the SDU almost parallel with a 10 mm lateral spatial offset and are focused by the last beamline mirror (M_3_) onto the same sample spot over a focal length of 2000 mm. The particular way of splitting the beam results in two essentially oval shaped spots of the same size, which we determined to be of 13 *μ*m diameter (semi-major axes). This focusing geometry results in an only small angular offset of 0.15° between both beam components such that any geometrical pulse broadening effects can be neglected. Spatial overlap of pump and probe spots is controlled by imaging both on a phosphor screen on the sample holder by an *ex situ* CCD camera equipped with a microscope lens. The pump fluence is controlled by the attenuation of the X-ray beam in the krypton-filled gas attenuator in the front end of the FEL and a set of exchangeable, absorbing Aluminum filters F_1_.

In this scheme, the experimental temporal resolution is essentially limited by the X-ray pulse width. As an independent means to determine the pulse length, we used time-resolved electron beam phase space tomography. We could determine the pulse length to lie in a range between 10 and 50 fs (FWHM) resulting in a temporal experimental resolution between 14 and 70 fs (FWHM).

Electronic order in magnetite was detected at the (0 0 $\frac{1}{2}$)^35^ superstructure peak.^22,25,31^ The peak intensity was recorded in a two-circle UHV diffractometer chamber.^36^ For magnetite single crystals with (001) surface orientation, this reflection can be detected in specular reflection geometry under an incidence angle of ≈22° with respect to the sample surface. The used magnetite samples were synthetic single crystals, which were oriented and cut to a face size of typically 1.5 × 1.5 mm^2^ and which were cleaved *ex situ* before introducing them into the ultrahigh vacuum chamber with a base pressure of 10^−9^ mbar. During the measurements, the sample temperature was held at 80 K. The free electron laser FLASH operated at a macro-bunch repetition rate of 10 Hz and a micro-bunch rate of 100 kHz with 30–40 micro-bunches per macro-bunch. For detection, an avalanche photo-diode was used which was screened from fluorescence light in the visible range as well as from the pump pulse photons by a 400 nm thick Al foil (F_3_). The amplified fast diode signal was directly recorded with a 2 GHz sampling rate resolving each individual micro-bunch contribution.

## III. DATA ANALYSIS AND DISCUSSION

**FIG. 2.**
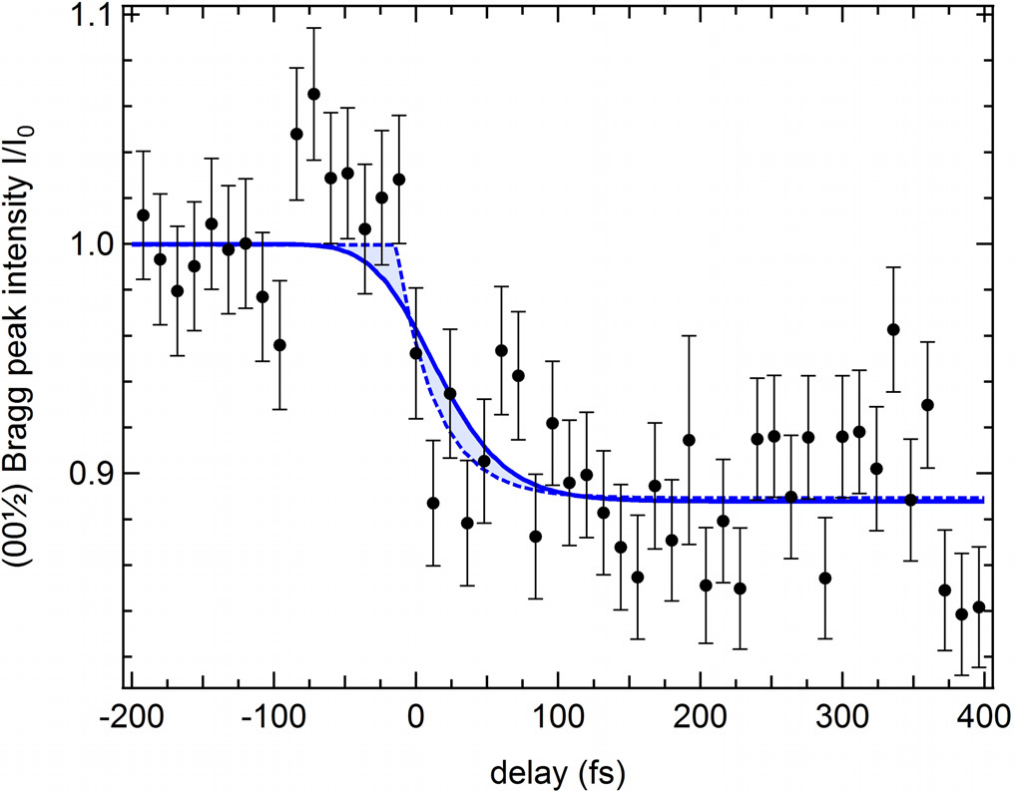
Time resolved resonant X-ray diffraction by two-color X-ray pump and probe: (a) Time resolved resonant X-ray diffraction from the (0 0 $\frac{1}{2}$) superstructure peak of magnetite after excitation by 177 eV photons. Before excitation, magnetite has been prepared in the low temperature, electronically ordered, insulating phase. The drop of the diffraction signal indicates a partial loss of electronic order. To estimate the decay time within the uncertainty range of the temporal resolution as determined from the pulse-length measurement (see Experimental Details), the data have been fitted by single exponentials by considering the range of possible temporal resolution (see the text). Assuming 10 fs resolution yields the dashed fit curve with a decay time *τ* = (28 ± 20) fs; the solid curve refers to an assumed temporal resolution of 70 fs resulting in *τ* = (30 ± 30) fs.

We now discuss the experimental results. Figure 2 shows the (0 0 $\frac{1}{2}$) diffraction peak intensity as function of the delay between pump and probe pulses. The error bars of the data points denote the experimental standard variation of the measured raw data for each delay point mostly due to fluctuations of the FEL. We observe a clear pump effect leading to a sudden drop in intensity. The shape of this drop is determined both by the intrinsic dynamics of electronic order and the temporal resolution. We analyzed the data by fitting a model of an exponential decay with time constant *τ*, convolved by a Gaussian of full width at half maximum of *w*, which is accounting for the experimental time-resolution. To deal with the uncertainty range of the experimental resolution of w≤ 70 fs, we varied *w* in this range; the resulting fit curves lie in the shaded region in Fig. 2. The best fit (minimum *χ*^2^) was achieved with a temporal resolution of unrealistic 10 fs (dashed line); a fit with the most conservative temporal resolution of 70 fs is shown by the solid line. Most importantly, the determined time-constant turns out to be largely independent of the precise time-resolution and is *τ* = (28 ± 20) fs and *τ* = (30 ± 30) fs for the above-mentioned 10 fs and 70 fs resolution, respectively.

**FIG. 3.**
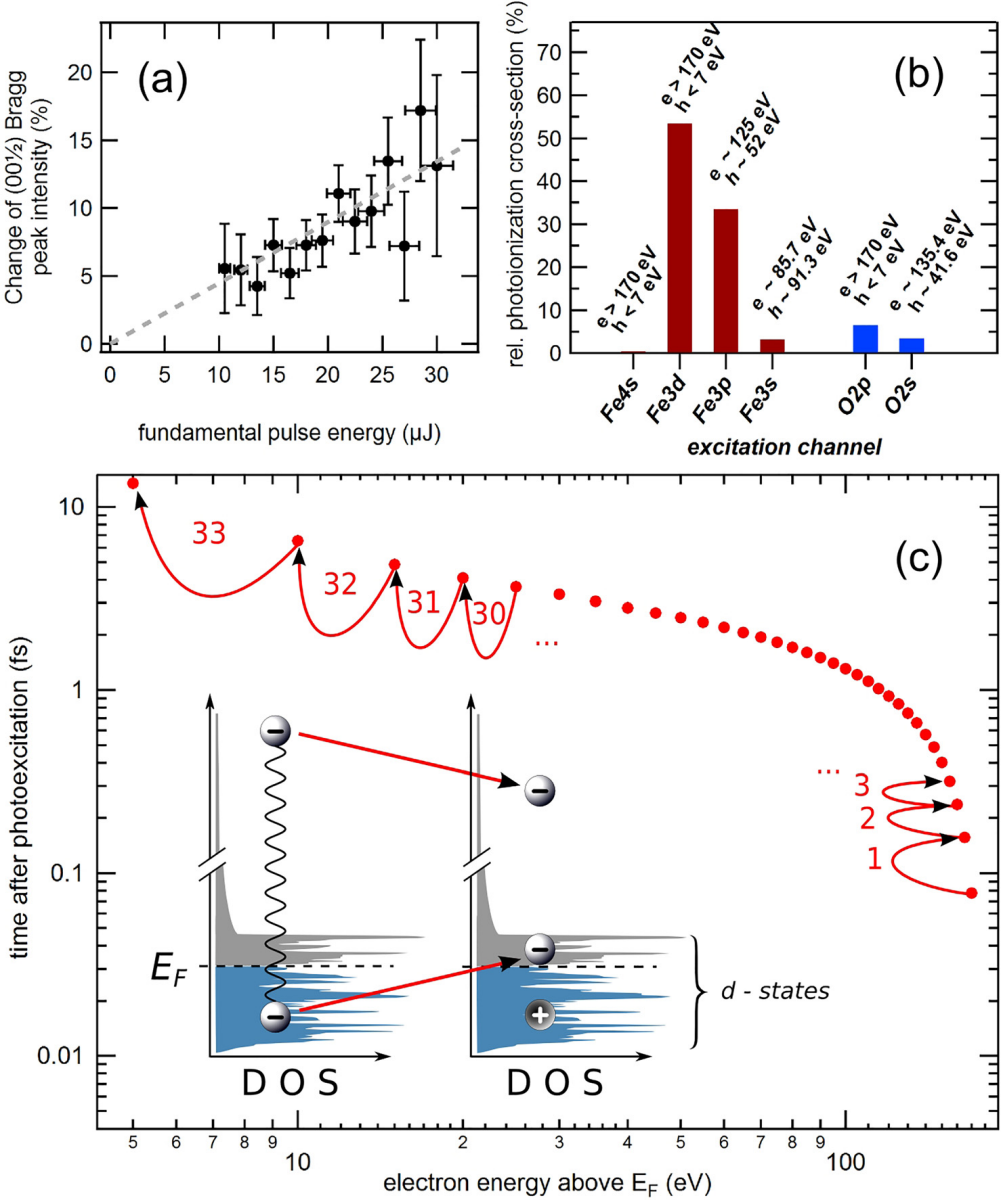
(a) Relative change of the (0 0 $\frac{1}{2}$) superstructure Bragg peak intensity for delays >100 fs as a function of fundamental pulse energy as measured by the gas monitor detectors. A linear dependence is observed. (b) Initial sub-shell photoexcitation in magnetite by photoabsorption of 177 eV photons. The excitation ratio of the particular electrons considers the individual atomic cross-sections,^39^ occupation number, as well as the stoichiometry. “e” and “h” correspond to the electron and hole energies, respectively, associated with the particular excitation process. (c) Ultrafast electron relaxation through inelastic scattering processes (impact ionization). The red symbols mark the excited electron energy after the 1st, 2nd, etc., inelastic scattering event by assuming an average energy loss of ≈5 eV per event.

In earlier experiments, the initial excitation occurred via an infrared laser pulse of 1.5 eV photon energy, while in the present experiment the pump pulse had a more than 100-times higher photon energy. In order to relate our finding to the earlier infrared-pump results, we analyze in the following the initial excitation scenario. We first address the fluence dependence: The initial drop of the (0 0 $\frac{1}{2}$) diffraction intensity depends linearly on the absorbed pulse energy, i.e., on the excitation density. This is shown in Fig. 3(a), where the pump effect [relative difference in signal between negative (< −100 fs) and positive delays (>100 fs)] is plotted versus the fluence. Pumping occurs hence via a linear process.

From the atomic photo-ionization cross sections, we derive that 97% of all photo-excitations create electrons with energies between 125 eV and 170 eV above *E*_Fermi_ [Fig. 3(b)]. The excited electrons relax on a sub fs-time scale by inelastic electron-electron scattering, so called impact ionization^32,37,38^ [Fig. 3(c)]: Single scattering processes predominantly result in electron-hole pair creation within the density of valence states in the material. We estimate the average energy loss of the excited electron through one such process as half the *d*-band energy width (≈5 eV). Subsequently, further scattering events ensue. The red symbols in Fig. 3(c) present the elapsed time after the *n^th^* scattering event. The time between two scattering events is estimated from the electron mean-free-path given by the universal curve and from the electron kinetic energy (without effective mass corrections). Within this simple model, after about 33 scattering events (i.e., around ≈10 fs after the initial excitation), the energy of the excited electron drops below 5 eV and the cascade basically terminates: Most of the excitation energy of the pump pulse is converted into electronic valence excitations within substantially less than 10 fs.

This estimation is consistent with our present experimental result. The final excitation scenario associated with the loss of electronic order is very similar to that caused by infrared pulse excitation as described in Ref. 23: It is a result of local destruction of electronic order induced by secondary excitations. We note that the structural relaxation, which is expected to occur on times of ⪆105 fs and ⪆65 fs,^28,29^ is not observable in our data, e.g., as a second time scale. In an earlier experiment, we found that when probing the (0 0 $\frac{1}{2}$) peak at the Fe-*L*_2,3_ resonance, i.e., when sensitive to electronic order like in the present experiment, structural relaxation is visible as a shift of the peak position on the detector.^23^ Since in the present study we did not use a spatially resolving detector, we would expect to be only sensitive to electronic dynamics.

## IV. CONCLUSION

By using a novel experimental approach, we study a driven, short lived temporal non-equilibrium state of a functional transition metal oxide at a free electron laser. In a time-jitter-free two-color X-ray pump and probe experimental scheme, we achieved the required temporal resolution of a few tens of femtoseconds. Within this timescale after exciting magnetite by a soft X-ray pulse, we observe a fast quench of the orbital order. The electronic dynamics appears to be faster than the expected lattice response thus hinting at a dynamic decoupling of electronic and structural DOF. Mostly because of the scatter in the experimental data, the (one-sigma) uncertainty interval for the electronic dynamics time scale borders on what is to be expected for the fastest structural response. More experimental work is needed for a final conclusion; our experimental scheme shows how such studies can be designed. We further discuss the mechanism of X-ray pumping: Via a fast cascade of electron scattering events the X-ray pump pulse leads to quasi-instantaneous valence excitations similar to what is achieved by optical pumping. Our experiment identifies the time-scales on which dynamic decoupling in functional solids occurs and demonstrates an experimental scheme to address them. It hence opens up a way to develop new concepts for their description.
